# Atrial Tachycardia Associated With a Tachycardia-Induced Cardiomyopathy in a Patient With Systemic Lupus Erythematosus

**DOI:** 10.7759/cureus.11626

**Published:** 2020-11-22

**Authors:** Faryal Subhani, Intisar Ahmed, Adil A Manji, Yawer Saeed

**Affiliations:** 1 Cardiology, Aga Khan University, Karachi, PAK; 2 Medicine, Aga Khan University, Karachi, PAK; 3 Medicine/Cardiology/Electrophysiology, Aga Khan University, Karachi, PAK

**Keywords:** systemic lupus erythematosus, atrial tachycardia, ablation

## Abstract

Systemic lupus erythematosus (SLE) is an autoimmune disease that involves multiple organ systems. Cardiovascular involvement in SLE is well described in the literature. Cardiac arrhythmias associated with SLE include sinus tachycardia, atrial fibrillation, and atrial ectopy or atrial tachycardia. In this report, we present the case of a patient with SLE who was found to have focal atrial tachycardia that mimicked sinus tachycardia on a 12-lead electrocardiogram (ECG). She was inappropriately treated as a case of sinus tachycardia initially. But she did not respond to the treatment and developed tachycardia-induced cardiomyopathy despite being on antiarrhythmic medications. She subsequently underwent successful radiofrequency catheter ablation and her left ventricular ejection fraction (LVEF) recovered within three months after the ablation.

## Introduction

Systemic lupus erythematosus (SLE) is an autoimmune disease entailing the formation of immune complexes and autoantibodies. Although it affects multiple organs, cardiac involvement is one of the most significant manifestations, and cardiovascular diseases are the most common cause of death among these patients. Myriad forms of cardiac manifestations have been reported in the literature, including the presence of late potentials in signal-averaged electrocardiogram (ECG) [1]. Long QT syndrome and ventricular arrhythmias have also been described in association with hydroxychloroquine treatment, which may result in sudden cardiac death in these patients [2]. Supraventricular arrhythmias, especially focal atrial tachycardia, has not been commonly seen in association with SLE. Atrial tachycardia is usually treated with medications and hardly requires any invasive treatment. In cases where pharmacological treatment is found to be ineffective, radiofrequency catheter ablation is an appropriate intervention [3]. Here, we present a case of a patient with SLE who was found to have atrial tachycardia but was initially misdiagnosed and treated as a case of sinus tachycardia. She developed tachycardia-induced cardiomyopathy despite good antiarrhythmic medications and subsequently required radiofrequency ablation.

## Case presentation

**Figure 1:**
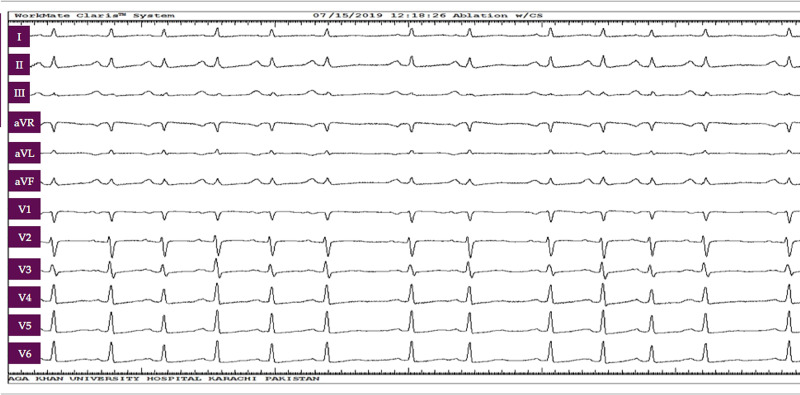
Initial 12-lead ECG Note the grouped P waves. Positive P waves in leads I and II and biphasic P wave in lead V1 suggest the origin of tachycardia to be near the sinus node (from the superior crista terminalis region of the right atrium or right pulmonary veins) ECG: electrocardiogram

A 42-year-old female patient with a six-year history of SLE, who had been taking hydroxychloroquine 200 mg and prednisolone 5 mg once daily, presented to the cardiology clinic with complaints of persistent palpitations and shortness of breath for the past six months. She did not experience syncope and denied chest pain. She did not have any family history of coronary artery disease, sudden cardiac death, and cardiomyopathy. On examination, she was found to be tachycardiac with a regularly irregular pulse and had no signs of heart failure. A 12-lead ECG showed tachycardia with an upright P wave in leads I and II, P wave morphology mimicking sinus P wave with a unique pattern of grouped P waves followed by a pause, which was again followed by a run of tachycardia (Figure 1).

Her echocardiogram showed normal-sized left and right ventricles with normal left ventricular ejection fraction (LVEF), no valvular abnormalities, and no pericardial effusion.

Initially, she had been treated at her community hospital as a case of sinus tachycardia, which is commonly seen in patients with SLE. She had been started on ivabradine and bisoprolol but her condition had not improved and she had continued to have symptoms despite being administered the maximum tolerable dose of the aforementioned drugs. She had then been referred to the local electrophysiology team who diagnosed the focal atrial tachycardia and she had been started on amiodarone and later on, she had undergone direct current cardioversion. However, despite all the above treatments, she had continued to have symptoms. She eventually presented to our hospital where a 12-lead ECG was consistent with focal atrial tachycardia, likely originating from the superior region of crista terminalis or right-sided pulmonary veins (Figure 1).

She was advised to undergo an electrophysiological (EP) study and ablation procedure because of her incessant tachycardia that did not respond to medications. She initially declined and decided to continue medical treatment. She returned after six months and reported worsening of symptoms. This time, she also reported New York Heart Association (NYHA) class II-III heart failure symptoms. Her repeat echocardiogram revealed a drop in LVEF from 60% to 25%. After extensive counseling, she finally agreed to undergo an EP study and ablation procedure.

**Figure 2:**
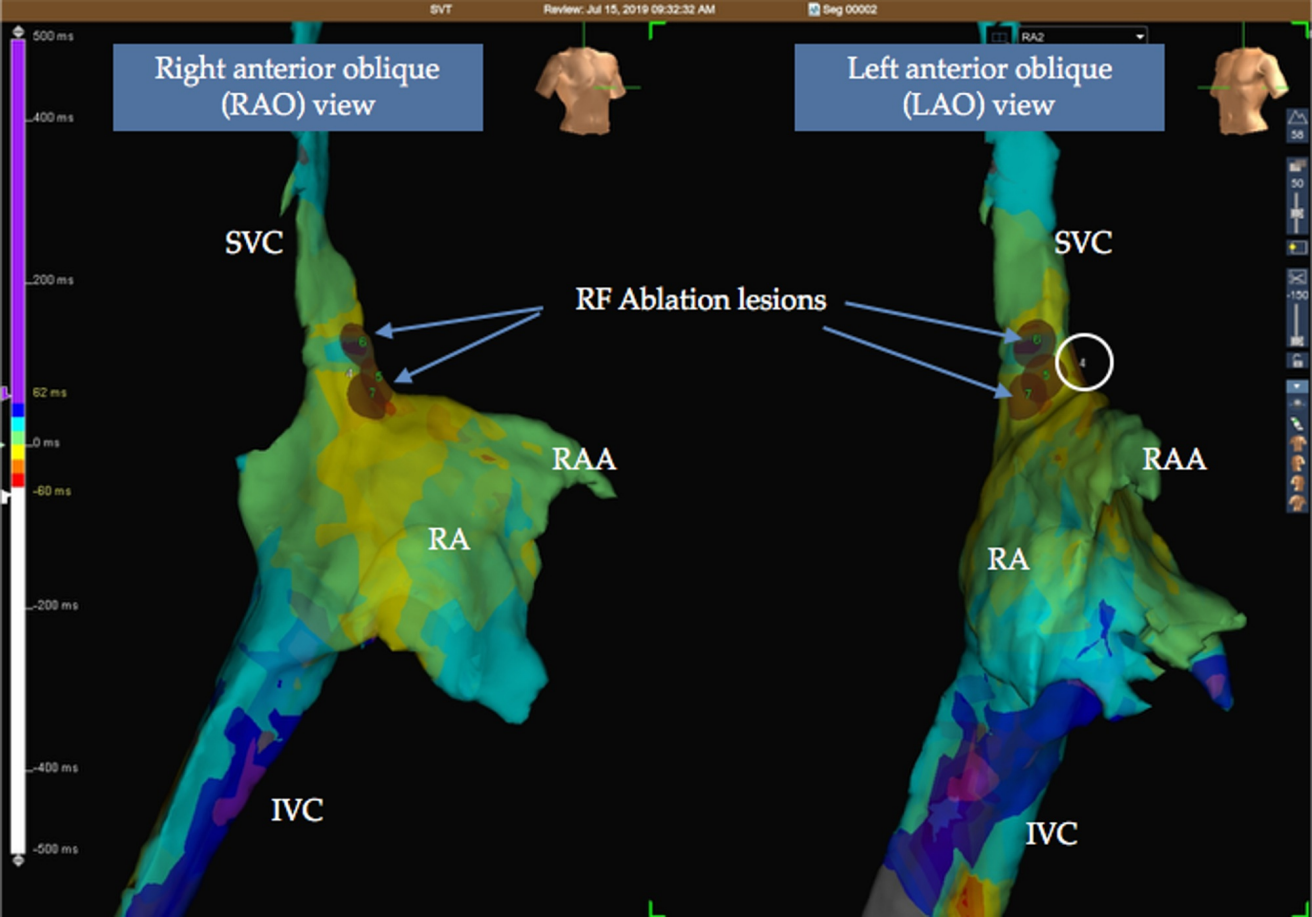
Right-atrium three-dimensional electroanatomical local activation time mapping with the EnSite Precision™ system Colors represent the activation sequence and centrifugal spread across the right atrium (white color represents the earliest activation site followed by red, yellow, green, and blue). The earliest signal is seen at site 4 circled with a white circle. Ablation at this point resulted in the termination of tachycardia SVC: superior vena cava; IVC: inferior vena cava; RA: right atrium; RAA: right atrial appendage; RF: radiofrequency

The EP study was performed with the EnSite Precision™ cardiac mapping system (St. Jude Medical/Abbott Inc, St. Paul, MN). A three-dimensional local activation map was created with a high-density catheter (Livewire™ Duo-Decapolar, 2-2-2, St. Jude Medical). The mean heart rate was 132 beats/min. The tachycardia had an extremely variable cycle length (406-616 msec). Both right- and left-sided atria were mapped, with activation mapping the location of tachycardia confirmed at the superior crista terminalis region in the right atrium (Figure 2).

**Figure 3:**
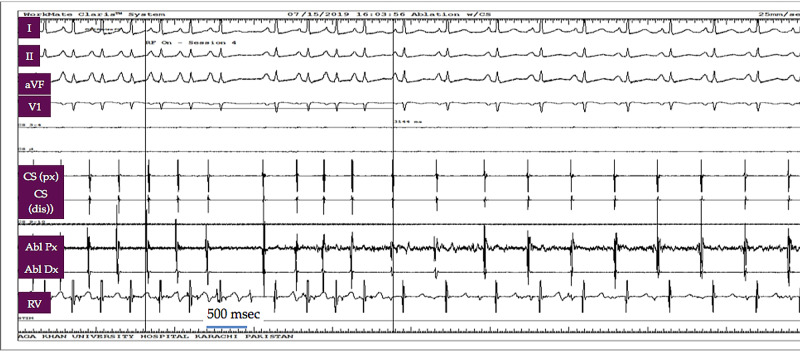
Electrophysiology study recording Radiofrequency ablation results in the termination of arrhythmia within 3.6 seconds (power: 30 W, temperature: 60 °C) CS: coronary sinus; Abl: ablation; Px: proximal; dis: distal; RV: right ventricular

Radiofrequency catheter ablation at this point resulted in the termination of atrial tachycardia (Figure 3).

**Figure 4:**
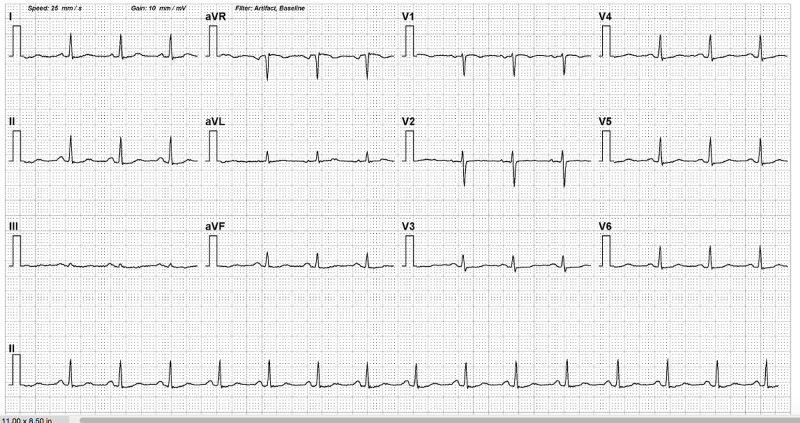
12-lead ECG at one-year follow-up ECG at one year after ablation reveals normal sinus rhythm ECG: electrocardiogram

Following an ablation procedure, the patient remained well and she was discharged the next day. Her repeat echocardiogram after three months of ablation showed a remarkable improvement in the LVEF, from 30% to 60%. She remained in normal sinus rhythm and asymptomatic at the one-year follow-up (Figure 4).

## Discussion

The pathogenesis of cardiac manifestations, especially arrhythmias, in SLE is reasonably well known [1]. Histological changes seen within the myocardium during initial phases of SLE include inflammatory cell infiltrates. With disease progression, myocardial necrosis, fibrotic replacement, and myocardial scaring are also observed [4]. Extensive myocardial fibrosis has been seen in the association of atrial fibrillation in patients with SLE with a cardiac MRI scan [5]. We can presume that these changes can result in atrial as well as ventricular arrhythmias in SLE patients. Common arrhythmias observed in SLE include sinus tachycardia, atrial ectopic beats, and atrial fibrillation, with sinus tachycardia occurring in 50% of all patients [6].

Atrial tachycardia is a type of supraventricular tachycardia and, based on the mechanism, propagation, and location of the electrical impulse, it can be identified as focal or macro-reentrant. It can also be the initiating trigger in atrial fibrillation. Focal atrial tachycardia accounts for around 10% of supraventricular tachycardias, which is characterized by a centrifugal spread of the wave of depolarization from a distinct focus within the atrial tissue and does not require another part of the conduction system for its propagation. It may be unifocal, which originates from one site due to reentry or enhanced automaticity, whereas multifocal atrial tachycardia has multiple sites of origin in atria and is characterized by the presence of isoelectric periods between P waves on an ECG. Multifocal atrial tachycardia is often associated with a history of pulmonary disease [7]. Although focal atrial tachycardia itself is the least common of these, the most common site of origin is the crista terminalis, specifically the mid and superior crista terminalis [8]. Other common foci in the right atrium include the ostium of the coronary sinus, para-hisian region, and the tricuspid annulus. In the left atrium, it most commonly arises from the ostia of the pulmonary veins. The left atrial appendage, the left-sided septum, and the mitral annulus are less common sites [9].

Our patient had several important features that made her case unique. Firstly, it is less common to see focal atrial tachycardia in a patient with SLE. That is why her rhythm was initially misinterpreted as sinus tachycardia. This potentially can have deleterious consequences as happened in our patient, although SLE may be only an association with the atrial tachycardia rather than the causative factor here. However, the presence of atrial tachycardia with multisystem autoimmune disease should make one wonder about cardiac involvement and lead to a prompt referral. Secondly, the tachycardia cycle length is variable with grouped P waves followed by the pause, which raises the possibility of micro-reentry and the presence of a myocardial scar. Thirdly, the occurrence of cardiomyopathy with heart rate varying from 110-130 in a young female should make us question whether this is only associated with SLE. A cardiac MRI could potentially answer the presence of a scar or myocardial involvement within the atria. Resolution of LVEF on the restoration of sinus rhythm with radiofrequency ablation points towards atrial tachycardia-induced cardiomyopathy. We still believe that these patients with autoimmune multisystem disorders need careful long-term follow-up as they can potentially develop further cardiac manifestations.

## Conclusions

In conclusion, focal atrial tachycardia cases should be distinguished from sinus tachycardia and, if not treated properly, they may lead to tachycardia-induced cardiomyopathy especially in patients with SLE or other multisystem autoimmune disorders. These patients can be provided with potentially life-saving treatments in the form of EP study and ablation. Prompt recognition and appropriate timely referral can prevent significant mortality and morbidity in these patients.
